# Insights into the Explicit Protective Activity of Herbals in Management of Neurodegenerative and Cerebrovascular Disorders

**DOI:** 10.3390/molecules27154970

**Published:** 2022-08-04

**Authors:** Tapan Behl, Rashita Makkar, Aayush Sehgal, Neelam Sharma, Sukhbir Singh, Mohammed Albratty, Asim Najmi, Abdulkarim M. Meraya, Simona Gabriela Bungau

**Affiliations:** 1 Chitkara College of Pharmacy, Chitkara University, Rajpura 140401, Punjab, India; 2 Department of Pharmaceutics, MM College of Pharmacy, Maharishi Markandeshwar (Deemed to Be University), Mullana-Ambala 133207, Haryana, India; 3 Department of Pharmaceutical Chemistry and Pharmacognosy, College of Pharmacy, Jazan University, Jazan 45142, Saudi Arabia; 4 Pharmacy Practice Research Unit, Department of Clinical Pharmacy, College of Pharmacy, Jazan University, Jazan 45124, Saudi Arabia; 5 Department of Pharmacy, Faculty of Medicine and Pharmacy, University of Oradea, 410028 Oradea, Romania; 6 Doctoral School of Biomedical Sciences, University of Oradea, 410028 Oradea, Romania

**Keywords:** neurodegenerative disorders, natural products, bioactive compounds, Parkinson’s disease, Alzheimer’s disease, Huntington’s disease

## Abstract

The longstanding progressive neurodegenerative conditions of the central nervous system arise mainly due to deterioration, degradation and eventual neuronal cell loss. As an individual ages, the irreversible neurodegenerative disorders associated with aging also begin to develop, and these have become exceedingly prominent and pose a significant burden mentally, socially and economically on both the individual and their family. These disorders express several symptoms, such as tremors, dystonia, loss of cognitive functions, impairment of motor activity leading to immobility, loss of memory and many more which worsen with time. The treatment employed in management of these debilitating neurodegenerative disorders, such as Parkinson’s disease (which mainly involves the loss of dopaminergic neurons in the nigrostriatal region), Alzheimer’s disease (which arises due to accumulation of Tau proteins causing diffusive atrophy in the brain), Huntington’s disease (which involves damage of striatal and spinal neurons, etc.), have several adverse effects, leading to exploration of several lead targets and molecules existing in herbal drugs. The current review highlights the mechanistic role of natural products in the treatment of several neurodegenerative and cerebrovascular diseases such as Parkinson’s disease, Alzheimer’s disease, ischemic stroke and depression.

## 1. Introduction

The neurodegenerative diseases display a diversity of longstanding progressive conditions of the central nervous system which are mainly triggered by the degradation and subsequent neuronal cell loss and manifest an ample number of symptoms including dystonia and tremors in the affected individuals followed by impairment in memory of the patients and damage to their cognitive functions such as their ability to talk, breathe, and walk. This leads to the gradual progression of the disease over time [1]. The factors mainly accountable for the initiation and prolongation of neurodegenerative disorders includes neuroinflammation, oxidative stress, mitochondrial dysfunction, altered misfolding of proteins and dysfunctional protein agglomeration accompanied with several other biological processes and environmental factors. These factors play a major role in the pathophysiology of several neurodegenerative disorders and simultaneously implicate their role in further progression of the diseases [2]. Several studies are underway to explore pathways which could combat neurodegenerative disorders by potentially targeting pathophysiological factors and aim at synthesizing medications which are highly efficacious and pose great benefit to the affected population with minimum adverse effects [3]. Several strategies and associated mechanistic pathways exhibited by various products are being studied, which further interact with the pathophysiological processes to induce neuroprotective effects by interrupting and postponing neurodegenerative processes. Since time immemorial, humans have been using natural products and compounds from natural origin for treatment of several chronic disorders in lieu of their medicinal properties. The natural plants and their bioactive components are being extensively researched in the recent years and are being further analyzed for their biological activity, nutritional properties, therapeutic benefits, and their role in providing potent health friendly therapies [4]. Several studies conducted in recent decades have significantly confirmed the neuroprotective effects of numerous natural plant-based products and bioactive molecules in the treatment of a variety of chronic conditions including cardiovascular disorders, metabolic disorders, endocrinological disorders, autoimmune diseases, neurological disorders, cancers etc. The natural herbal drugs and bioactive compounds isolated from them have emerged as a magical treatment in the management of neurological disorders and have outshined in lieu of their neuroprotective abilities [5,6]. The current review highlights the therapeutic potential of a number of natural products and their isolated bioactive compounds in the management of neurodegenerative and cerebrovascular diseases and their effect on neuro-pathophysiological conditions.

## 2. Potential Therapeutic Molecules of Natural Products against Neurodegenerative and Cerebrovascular Diseases

**Figure 1:**
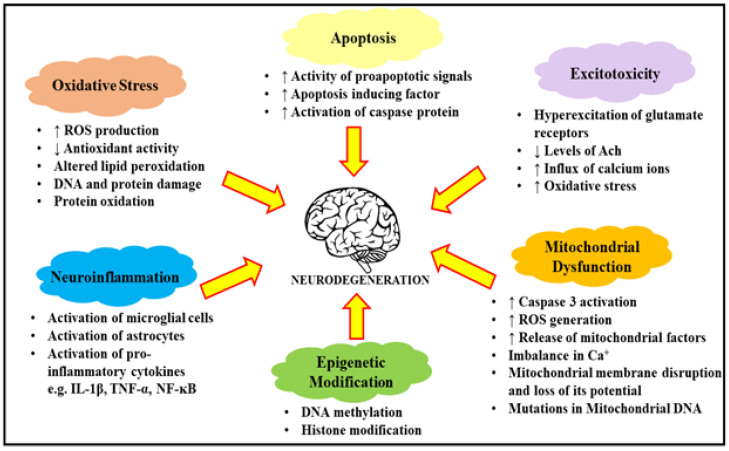
Description of factors leading to neurodegeneration processes. The further progression of these processes leads to the exacerbation of apoptotic factors, the increase in oxidative stress and excitotoxicity, neuroinflammation and mitochondrial dysfunction. ROS: reactive oxygen species; DNA: deoxy ribonucleic acid; Ach: Acetylcholine; IL-1β: interleukin 1 β; TNF-α: Tumor necrosis factor-α; NF-κB: Nuclear factor kappa B.

Researchers have studied and investigated the mechanism of neuronal damage and neuronal cell death from molecular level to the organ level for several years. The accretion of neurotransmitters (predominantly glutamate) in the tissues of the brain can lead to unwarranted injury in the brain which further induces the overstimulation of the nerves and neuronal cells and triggers the death of neuronal cells [7]. As per World Health Organization (WHO) data from 2012, over 35.6 million individuals globally are affected by dementia, with Alzheimer’s disease accounting to over 60–70% of this affected dementia population [8]. The pathophysiology of Alzheimer’s disease is not completely elucidated and is still being investigated. The utmost prevalent type of Alzheimer’s disease i.e., the late onset sporadic Alzheimer’s disease, is responsible for the vulnerability in genetic makeup of the individual and its corresponding environmental factors [9]. The natural products and bioactive compounds of natural origin have recently gained popularity as an alternative therapeutic approach in management of not only Alzheimer’s disease but also certain associated neurodegenerative disorders. The neuropathophysiological characteristics of Parkinson’s disease involves intracellular protein aggregate accumulation, Lewy bodies and Lewy neuritis, and aggregated and mistreated forms of α-synuclein proteins combined with the persistent loss of dopaminergic neurons from the nigrostriatal region of the brain [10]. Huntington’s disease, furthermore, is a hereditary dominant autosomal neurodegenerative disorder which is chiefly manifested by disturbance in motor functioning, loss of cognition, and mental abnormalities. The disease is associated with an unstable expansion of cytosine-adenine-guanine (CAG) in the Huntington gene present on chromosome 4 [11]. Several biological factors such as increased reactive oxygen species and oxidative stress, neuroinflammation, dysfunctional mitochondria and epigenetic modifications play a chief role in neuro pathophysiology and are significantly involved in the initiation and aggravation of neurodegenerative disorders, as described in Figure 1 [12]. The role of oxidative stress has been found to be prominent in the aggravation of Parkinson’s and Alzheimer’s disease besides other associated neurodegenerative disorders. The generated free radicals due to oxidative stress attack the neuronal cells and have a significant involvement in calamitous neurodegradation. Oxidative stress in the individuals is mainly caused by an imbalance in the generation of free radical species and the defensive activity of antioxidants, leading to cellular damage including impairment in the DNA and altered mitochondrial functioning [13]. The generation of amyloid-β proteins in individuals suffering from Alzheimer’s disease and their further aggregation leads to the phosphorylation of tau proteins and to pathophysiological changes. The adaptive and innate immune system of the central nervous system are also indulged in the neuroinflammatory pathways and are directly connected with neurodegeneration and can directly lead to neuroinflammation [14]. Microglia cells in the central nervous system are the chief component of the innate immune responses and cause morphological changes in response to pathological alterations. Upon activation, the microglial cells release numerous inflammatory and pro-inflammatory mediators like cytokines, chemokines and various cytotoxic molecules [15]. The inflammatory mediators released further permit astrocytes to retort the survival and reparation of secondary inflammatory or growth factor repair responses. The mitochondrion, as the site responsible for oxidative phosphorylation, aids in the maintenance of low cytosolic calcium ion concentrations, since the excessive absorption of calcium ions leads to a fall in the functioning of the mitochondrial membrane and causes the opening of mitochondrial pores [16]. The epigenetic modifications such as the methylation of DNA and histone protein modifications also contribute majorly to the onset of neurodegenerative diseases. The epigenome is mainly accountable for the 3D structure of the genomic constituents within the nucleus and its molding. It acts as a bridge between the environment and the genetic presentation of an individual and plays a major role in the etiology of several complex neurological diseases. The changes in epigenomes lead to further alterations in the genetic expression and change the cellular phenotype in a heritable manner. The most extensively studied modification in epigenomes includes DNA methylation and further alterations in the histone proteins. The addition of a methyl group at a cytosine nucleotide of DNA leads to DNA methylation, which further influences the performance of DNA by stimulating/repressing several transcriptional factors and modifying genetic activity, thereby modulating the cellular functioning of the individual. The modifications in histone proteins such as acetylation and methylation of lysine residues on their tails post-translation are also a common form of epigenetic modification and directly influence the expression of genes by producing alterations in the chromatin structure. Several studies have shown significant improvement upon treatment with histone deacetylase (HDAC) inhibitors in AD patients implying the evident role of epigenetic modifications in the development of neurodegenerative diseases. HDAC is a key enzyme which is majorly involved in the acetylation of histone proteins, and its inhibitors have been found to significantly inhibit α-synuclein toxicity in the dopaminergic neurons of patients suffering from Parkinson’s disease.

## 3. Natural Products and Their Protective Role in the Management of Alzheimer’s Disease

Alzheimer’s disease is a highly complex neurodegenerative disease and is globally one of the most common causes of dementia. More than 50 million patients were been reported to suffer from Alzheimer’s disease globally according to the World Alzheimer Report in 2019. The disease occurs due to multifactorial reasons which contribute to its pathophysiological complexity [17]. Oxidative stress, a decrease in neurotransmitter levels in brain (eg.Butylcholine, acetylcholine), neuroinflammation, the accumulation of amyloid-β (Aβ) proteins and their further formation of plaques and high monoamine oxidase activity are amongst the major pre-neurodegenerative factors [18]. For effective treatment of neurodegenerative disorders, a quick diagnosis of the condition is necessary, the failure of which leads to further non-specific complications. The initial symptoms of stage one Alzheimer’s disease include disturbances in memory and chronic tiredness, while the middle and later stages express symptoms that are more characteristic to neurodegradation [19]. The patients experience significant loss in memory with disturbances in their learning abilities along with insomnia. Recently, drugs of natural origin have become very popular for the treatment of Alzheimer’s disease and are being actively explored. The natural region drugs have inexhaustible sources and comprise of plethora of active components which have been used for their therapeutic properties in the treatment of numerous disorders [20]. The plants and their metabolites rich in terpenes and polyphenols have been widely explored due to their antioxidant, anti-inflammatory, sedative, anti-bacterial and enzyme modulating biological activities.

**Figure 2:**
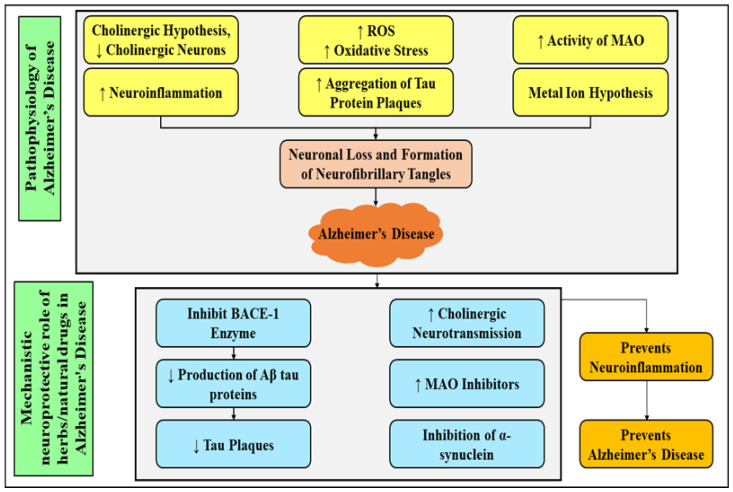
Pathological factors responsible for onset of Alzheimer’s disease. BACE-1: β-secretase; MAO: mono amino oxidase; ROS: Reactive oxygen species.

The herbal drugs that are used in the management of Alzheimer’s disease function by activating the neurotransmission of cholinergic neurons, promoting the inhibition of BACE1, α-synuclein and monoamine oxidase (MAO) proteins and preventing neuroinflammation are described in Figure 2 and Table 1.

### 3.1. Activation and Targeting of Cholinergic Neurotransmission

The cholinergic system of the human body controls several cognitive body functions including memory and learning. The neurons within the cholinergic system are a major source of innervation in the hippocampus and cortex regions. These neurons release O-acetyltransferase (ChAT), which catalyzes the transmission of the acetyl group from the coenzyme acetyl-CoA to choline to produce acetylcholine (ACh) [21]. The enzymes acetylcholinesterase (AChe) and butyrylcholinesterase (BuChe) hydrolyze the neurotransmitter ACh back to choline, thereby maintaining a balance of neurotransmitters in the system. It has been proposed by the cholinergic hypothesis that a downfall in the neurotransmission of cholinergic neurons and their loss in patients with Alzheimer’s disease may produce cognitive defects. Besides a drop in the level of ChAT mRNA expression in the brain of Alzheimer’s patients, its activity is also decreased, which is asynchronous with synaptic loss. For decades, the research on AChE inhibitors has been an area of interest for its role in the management of Alzheimer’s disease, and most of the drugs approved by Food and Drug Administration are AChE inhibitors [22]. However, unfortunately, the clinical studies undertaken have demonstrated that the therapeutic potential of AChE inhibitors in the management of Alzheimer’s disease did not perform as expected. The brains of patients suffering from Alzheimer’s disease comprise very high levels of BuChe, hence several anti-Alzheimer’s drugs target the cholinergic neurons, including BuChe inhibitors, and promote the expression of ChAT, thereby producing protective effects by stimulating nerve growth factors and brain derived neurotrophic factors and their receptors. The aggregation of Aβ plaques is associated with both AChE and BuChE enzymes. AChE increases the production of Aβ peptide fibril aggregates and leads to the formation of Aβ-AChE complexes [23]. Usually, the activity of AChE is reduced in the brain of patients suffering with Alzheimer’s disease, but his concentration is enhanced after binding to the plaques of Aβ. Therefore, both the enzymes AChE and BuChe are being abundantly explored for their activity in the management of Alzheimer’s disease. The extracts of several medicinal plants belonging to family Rutaceae, Ranunculaceae and Berberidaceae have been found to be rich in isoquinoline alkaloids, which have a tendency to effectively inhibit the activity of enzyme AChE and have thus been found to be effective in the management of Alzheimer’s disease. Furthermore, the herbal extracts of plants, namely *Coptis chinensis*, *Berberis bealei* and *Phellodendronchinense*, have also been characterized as possessing isoquinolone alkaloids such as coptisine, berberine and palmatine, in higher concentrations, and thus possess a substantial ability to constrain ACh via synergistical enhancement. The study also revealed that the aforementioned active extracts are not cytotoxic in nature at doses which can significantly limit the activity of AChE. Apart from the aforementioned natural products and their extracts, several other natural product preparations and extracts chiefly used in Chinese medicines have also demonstrated their therapeutic activity in the management of Alzheimer’s disease by altering the expression of nerve growth factors and brain-derived neurotropic factors and their related receptors [24,25,26]. The bio active compounds derived from ginger have also been shown to increase the levels of nerve growth factors and improved the impaired memory in scopolamine induced animal models of dementia. Transhinone II, by active compound isolated from the extracts of *Xanthocerassorbifolium*, has been studied for its ability to save the spines of dendrites on neuronal cells by transducing signals through brain derived neurotrophic factor and impose improvement of cognitive conditions in animal models of Alzheimer’s disease. The active molecule was also found to be helpful in promoting the synthesis of brain derived neurotrophic factors by inducing depolarization. A natural product named *Huperzia serrata* was also found to inhibit the activity of the enzyme AChE in mice models of Alzheimer’s disease and further ameliorated the impairment of cognition. Therefore, several natural products have been found to successfully diminish the symptoms of Alzheimer’s disease and can be used extensively in the management of this doubly debilitating neurodegenerative disorder [27].

### 3.2. Stimulation of Inhibitory Activity of BACE-1

The accumulation of Aβ plaques is one of the most studied and explored factors responsible for neurodegeneration in Alzheimer’s disease, as it plays a prominent role in the clustering of neurotic senile plaques. The Aβ peptides are discharged outside of cells in healthy individuals and are further removed and excreted. The process is disrupted with aging, and the ability of an individual to offload amyloid compounds is reduced, which leads to its accumulation in the brain in the form of aggregates and plaques. Extensive research has led to a thorough understanding of the creation of Aβ peptides and their structures and their influence on the functioning of the brain [28]. The Aβ peptides are created in two stages. During the first stage the amyloid precursor protein (APP) causes the splicing of enzyme β-secretase or BACE-1. This step causes the production of soluble amyloid precursor proteins (sAPPβ) and a 99 amino acid fragment which for the produces 38–43 amino acid peptide compounds and APP intracellular domain (AICD) upon action of another enzyme, namely γ-secretase. The Aβ 40 and Aβ 42 are the main products created and pose the greatest threat in initiating Alzheimer’s disease, as they play a crucial role in the creation of senile plaques [29]. The Aβ 42 is highly predisposed towards aggregation and is the most toxic form of Alzheimer’s disease. The BACE-1 enzyme can prudentially determine the amount of Aβ produced and you can also persuade the overproduction of toxic Aβ 42. Hence, the BACE-1 enzyme has been found to possess an evident role in the management of enzymes disease and several studies are being conducted on natural products which help in the management of this debilitating neurodegenerative disease by acting through the BACE-1 enzyme [30]. Several BACE-1 inhibitors have been tested in the past in phase II and phase III clinical trials such as verubecestat, lanabecestat, atabecestat, elenbecestat and umibecestat. The selected doses from the mentioned BACE-1 substances significantly reduced Aβ in the cerebrospinal fluid by 90%. Several studies have demonstrated that a therapeutic window for BACE-1 inhibitors is necessary to be evaluated prior to the deposition of Aβ plaques sufficiently to embark on a preventive therapy. The efficacy of preventive therapy depends upon the duration of the administration of drug end its further monitoring to minimize the associated side effects. Both clinical and nonclinical studies presented a range of adverse effects upon the administration of BACE-1 inhibitors such as retinal toxicity in animals, hepatotoxicity in humans, cognitive impairment, weight loss, sleep disturbances, anxiety, and suicidal ideation [31]. Therefore, to achieve an efficacious and safer therapeutic effect, selecting an optimum dose is a milestone in itself. The current subsection explains the in vitro, in vivo, and in silico studies performed for natural products and bioactive molecules derived from them in the management of Alzheimer’s disease via the inhibition of BACE-1 enzymes.

### 3.3. Inhibition of α-Synuclein

Based on ample data collected from several studies to understand the pathogenesis of neurodegenerative disorders including Alzheimer’s disease, an evident correlation between the dysfunction of neuronal mitochondria and the disease initiation has been confirmed. The accumulation of α-synuclein protein abnormally disrupts the normal functioning of neuronal mitochondria and induces an increase in oxidative stress and the degeneration of neurons. This low molecular weight protein has an ability to release proinflammatory cytokines such as nitric oxide and reactive oxygen species upon activation of microglia which results in neuroinflammation and further neuronal death. Synucleinopathic diseases involve the accretion of molecules rich in α-synuclein proteins [32]. Oligomers, fibrils end protofibrils are some types of accumulate which are synthesized during α-synuclein protein aggregation which may be neurotoxic in nature and initiate neurodegeneration. Therefore, the compounds that possess the ability to target the accumulation of this low molecular protein have emerged as a promising therapeutic approach in preventing the progression of Alzheimer’s disease. Epigallocatechin-3-gallate (EGCG), a natural origin bio active compound, inhibited the fibrillogenesis of α-synuclein protein by binding to its natively unfolded polypeptides at the C-terminus site directly and preventing their transformation into toxic aggregated intermediate compounds as revealed by computational molecular docking analysis. The EGCG also promoted the synthesis of nontoxic unstructured α-synuclein proteins suggesting their neuroprotective role by altering the aggregatory pathways involved in Alzheimer’s disease [33,34]. Another natural origin bioactive compound, namely protocatechuic acid at 10, 20, 15 and 100 umol/L doses significantly inhibited the aggregation of Aβ and α-synuclein proteins by disturbing the stability of prefabricated fibrils and preventing cell death.

**Table 1:** Herbal drugs along with their mechanisms in the management of Alzheimer’s disease.

Name of the Plant	Target of Action	Remarks	Ref.
*Piper nigrum* (Black Pepper)	AchE, CRP, NF-ĸB	Increased levels of Ach in brain Decreased levels of serum inflammatory cytokines in brain Prevents neuroinflammation and promotes cholinergic neurotransmission	[35]
*Foeniculum vulgare* (Fennel)	AchE	Decreases the activation of AchE enzyme in brain Prevention of amnesia	[36]
*Ocimum sanctum* (Tulsi)	AchE	Decreases the activation of AchE enzyme in cortex, medulla, cerebellum and mid-regions of the brain Prevention of cognitive behaviour	[37]
*Lavandula angustifolia* Mill. (Lavender)	AChE, Matrix metalloproteinase, ROS	Decreases the activation of AchE enzyme in brain Increased antioxidant activity Decreased production of reactive oxygen species and nitric oxide Overall improvement in cognitive behaviour	[38]
*Olea europaea* (Olive)	MDA, NO, COX-2	Decreases the production of reactive oxygen species, malondialdehyde and nitric oxide Minimizes oxidative stress	[39]
*Camellia sinensis* (Green tea)	AChE, COX	Improved cholinergic functions	[40]
Ajmalicine	BACE-1	Inhibits the activity of BACE-1 enzyme by binding to its catalytic site	[41]
Berberine	BACE-1	Inhibits the activity of BACE-1 enzyme non-competitively by binding to methylenedioxy group at the D ring of the enzyme	[42]
Gallic acid	BACE-1	Improved learning and memory Amelioration of cerebral amyloidosis	[43]
Epigallocatechin-3-gallate	α-synuclein	Inhibits the aggregation of α-synuclein protein and prevents further accumulation of Aβ-plaque	[44]
*Hypericum afrum*	MAO A and MAO B	Decreased production of amyloid plaques	[45]
*Cytisusvillosus*	MAO A and MAO B	Enhancement of dopaminergic neurotransmission	[45]
*Curcuma longa*	MAO A and MAO B	Strong MAO inhibition and thus prevention of further deterioration of the disease	[46]

## 4. Natural Products and Their Protective Role in Management of Parkinson’s Disease

**Figure 3:**
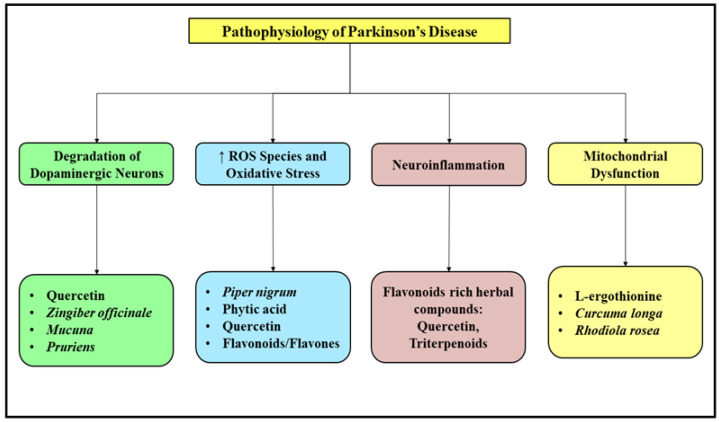
Pathological factors responsible for initiation and prolongation of Parkinson’s disease along with a list of herbal plants and bioactive natural origin molecules exhibiting anti-Parkinson activity. ROS: Reactive oxygen species.

Parkinson’s disease is an age associated neurodegenerative disorder associated with disability in locomotor activities which arises due to the gradual deterioration of dopaminergic neurons in the substantia nigra pars compacta region of the brain leading to substantial loss of striatal dopamine [47]. The treatment approach for this debilitating disorder mainly aims at replenishing the dopamine reserves and further preventing its synaptic degradation and lowering the levels of excess acetylcholine. The goal of pharmacotherapy for the management of Parkinson’s disease is administration of Levodopa or L-dihydroxy phenylalanine, which further produces several side effects such as fluctuations in motor activity accompanied with nonmotor complications and dyskinesia. To overcome these adverse events, several agonists of dopamine receptors and inhibitors of MAO B enzyme have also come in to use [48]. The effects produced by the consumption of standard anti-Parkinson medications are temporary, although they mitigate the symptoms of this debilitating disease but fail to slow down the progression of disease and hence lead to a dire need of exploration of alternative novel approaches to further improve the existing pharmacotherapy employed in management of this disease [49]. Several natural herbal based products are being tested and evaluated for their neuroprotective activities and further verify their role as either independent or as adjunctive therapy in the management of Parkinson’s disease. The natural products include extract obtained from herbal drugs and the further isolation of active ingredients from them and phytochemical molecules and their bioactive derivatives [50]. The treatment mainly aims at replenishing the degraded dopaminergic neurons, scavenging free radicals and reducing oxidative stress, preventing neuroinflammation and mitochondrial dysfunction. Figure 3 enlists some of the commonly used natural plants and their products used in the management of Parkinson’s disease.

### 4.1. Mucuna pruriens

*Mucuna pruriens*, commonly known as atmagupta or velvet bean, is a tropical legume mainly found in Africa, India, parts of the Caribbean, and in South and Central America. The plant has been known to possess antidepressant activities for some time. The seeds of the plant have been used in the treatment of Parkinson’s disease in India, as the seeds have been found to be rich in L-dopa, which has been successfully isolated [51]. Further phytochemical screening of the water and ethanolic extracts of the plant signified the presence of tannins, flavonoids, saponins, terpenoids, anthraquinones and cardiac glycosides which are responsible for its medicinal properties. The seeds are also rich in proteins, carbohydrates, lipids, minerals, sterols and fiber, and the precursor of dopamine, L-dopa, accounts for approximately 7–10% of the seed extract, indicating its crucial role in the management of Parkinson’s disease [52].

### 4.2. Nicotine

Nicotine is a naturally occurring alkaloidal compound which is mainly present in the tobacco plant. Nicotine has a tendency to penetrate the blood brain barrier readily and hence mimics the effects of acetylcholine by binding to nicotinic acetylcholine receptors (nAchR) which play a crucial role in the regulation of the dopaminergic function in the brain. A significant loss of nicotinic receptors with altered deficient secretion of dopamine has also been found in Parkinson’s disease, thereby implying a promising role of nicotine in the pharmacotherapy of Parkinson’s disease. The nAchR are extensively distributed throughout the brain, including the nigrostriatal pars compacta region, and exert a significant effect on several behavioral parameters associated with the disease. A significant decrease in the nAchR has been found in the nigrostriatal pars compacta region of the brain in patients suffering from Parkinson’s disease. The major symptom of Parkinson’s disease, dyskinesia, has also been found to be significantly reduced upon the administration of nicotine, which further stimulates nAchR and can be assessed as beneficial for the patients. Evidence from various clinical and experimental studies has suggested the attenuation of neurodegeneration and a great decrease in the amount of symptoms produced due to the onset of the disease. The incidence of Parkinson’s disease was found to be reduced by approximately 50% in patients consuming tobacco owing to its neuroprotective effects as per epidemiological studies [53,54]. The acute consumption of nicotine stimulates the functioning of nicotinic receptors, while excessive or chronic consumption leads to an elevation in the number of nicotinic receptors suggesting its possible role in the improvement and management of Parkinson’s disease.

### 4.3. Phytic Acid

The role of iron in the pathogenesis of Parkinson’s disease has been known for some time. The increase in the levels of iron in the brain interferes with the functioning of dopaminergic neurons and further interacts with several molecules such as dopamine, tyrosine hydroxylase and synuclein. The accumulation of iron in different regions of the brain increases the production of reactive oxygen species and further elevates oxidative stress in the brain via the Fenton reaction and produces neurodegeneration [55]. Targeting iron-induced reactive oxygen species and oxidative stress has emerged as a promising approach in the management of Parkinson’s disease. Phytic acid has been abundantly found in cereals, legumes, nuts, pollen, oil seed and spores and successfully inhibits the hydroxyl radical formation catalyzed by iron via iron chelation and prevents the oxidation of lipids. Phytic acid is considered as an antinutrient agent in lieu of its ability to chelate divalent materials and decrease their absorption which is noted as one of its beneficial properties. Several studies have reported the neuroprotective effects of phytic acid in preventing DNA damage and the neuronal death of dopaminergic neurons. Phytic acid is also a lipophilic molecule and readily penetrates the blood brain barrier, which further supports its neuroprotective property and hence its consumption serves therapeutic effects in the management of Parkinson’s disease. Phytic acid mainly acts by chelating iron accumulation by acting as a strong antioxidant, improves the uptake of glucose in the brain, and prevents the deposit of fat on the cerebral arteries and encourages normal functioning of the brain by improving oxygen and blood supply [56]. It also controls the influx of calcium in the cells of the brain and prevents dopaminergic neuronal cell death due to calcium overload.

### 4.4. Pepper

*Piper nigrum* and *Piper longum* are the most widely consumed species of the pepper plant for their therapeutic potential in the Ayurveda system of medicine. The fruit of Piper longum are rich in numerous alkaloids, piperine being the chief phytoconstituent, and are useful in the treatment of constipation, gonorrhea, diarrhea, tongue paralysis, malaria, cholera and viral hepatitis and is a good stimulant and carminative in nature [57]. It is also consumed to treat bronchitis and associated respiratory infections, cough, asthma and several splenic disorders. The topical application of pepper provides relief from muscular pain and inflammation, and it is also used as a sedative to treat epilepsy and insomnia. Lately, pepper has been extensively researched for its profound effects in the management of central nervous system disorders and is also reported to improve cognitive functioning in patients with Alzheimer’s disease. Piperine enhances the absorption and bioavailability of several drugs due to its effect on brush border of intestine and shows better neuroprotection against oxidative stress and cell death in dopaminergic neurons when administered with other medications in MPTP Parkinson models in mice [58].

### 4.5. Ginger

Ginger (*Zingiber officinale*) is among the most important spices consumed and belongs to the family of Zingiberaceae. It is mainly comprised of sesquiterpenoids, zingiberene being the major phytoconstituent, essential oils, and monoterpenoids such as citral and cineol. Gingerol is the major pungent principle of ginger and produces zingerone when cooked, and both of these phytoconstituents possess analgesic, anti-inflammatory, antipyretic, antibacterial and sedative properties. The plant has also been reported to possess antitumor activity and is effective against nausea, stomach ache, diarrhea, joint and muscle pain. Pretreatment with zingerone uplifts the levels of dopamine in the nigrostriatal region by increasing free radical scavenging due to the elevation of superoxide dismutase. Then by implying its protective role in management of Parkinson disease and its associated symptoms [59,60]. The plant, in combination with natural vitamin E or tocopherols, has been also studied for its curative effects in the management of bipolar disorder, attention deficit hyperactivity disorder and other neurodevelopmental diseases.

## 5. Natural Products and Their Protective Role in the Management of Depression

Depression is a serious medical neurological condition and is highly prevalent in both the developing and developed nations, with an estimated lifetime prevalence of over 15–20%. The most acceptable theory of researchers that facilitates the understanding of depression is the monoamine theory, according to which a decrease in monoamine neurotransmitters, namely dopamine, norepinephrine and serotonin, is found in the brain of the depressed patients. The antidepressant therapy mainly focuses on the reversal of monoamine neurotransmitter deficiency in the brain. Several new antidepressants such as venlafaxine and many more which promote the reuptake of neurotransmitters and prevent their degradation thereby improving the symptoms of depression have been introduced into the market [61,62]. However, long term consumption of these antidepressant drugs or their chronic use can induce serious adverse reactions upon their interaction with food and other concomitant drugs. Besides the availability of vast antidepressant drugs, there still exists over 10–30% of the population globally who still continue to experience episodes of depression, thereby suggesting the need to discover and develop newer agents with antidepressant properties and enhanced efficacy with minimal associated adverse effects [63]. Several ongoing studies have established natural polyphenol compounds isolated from a wide array of natural products which possess antidepressant properties and are being utilized as adjuvants in the management of conditions like major depression. The natural polyphenol molecules obtained from herbal drugs are non-essential micronutrients and possess several physiological and biochemical properties. They have been discovered to play a significant role in the management of different neurological and psychological disorders [64]. The polyphenol molecules exert a neuroprotective effect and significantly modulate the neurotransmission of monoaminergic neurons in the brain and have an anti-depressant-like activity. Some of the major polyphenol molecules with anti-depressant effects are described below.

### 5.1. Curcumin

Curcumin is a major phytoconstituent isolated from the legumes of the plant *Curcuma longa* and is renowned for its diverse biological activity and use in the Indian system of medicine. Curcumin efficiently modulates the activity of several neurotransmitters in the brain by regulating the levels of enzyme monoamine oxidase A (MAO A) and monoamine oxidase B (MAO B), and is studied to possess its role in the management of neurodegenerative disorders. In a study conducted in stressed mice, curcumin significantly promoted neurogenesis in the hippocampal region of the brain, and its effect mimicked the activity of imipramine, standard tricyclic antidepressant drug. The antidepressant effects of curcumin were also started in both rats and mice through forced swim test [65]. The levels of neurotransmitters serotonin and noradrenaline were also found to be increased upon consumption of curcumin in the hippocampal and frontal cortex region of the brain. In a study of olfactory bulbectomy-induced animal model of major depression, curcumin was found to significantly reverse the decreased levels of neurotransmitters, mainly serotonin and noradrenaline in response to the animal model of olfactory bulbectomy and increase the neurotransmission of neurotransmitters in the hippocampal region of the brain of the mouse. Curcumin is believed to exhibit antidepressant effects owing to its involvement and interaction with the serotoninergic receptors by modulating adenyl cyclase and the cAMP pathway. The release of another neurotransmitter, namely glutamate, is also inhibited by curcumin, which further displays its anti-depressant effects [66]. The glutamate-mediated decreased levels of brain derived neurotrophic factors (BDNF) were also reversed by curcumin, thereby promoting its neuroprotective effects and enhancing its anti-depressant-like activity.

### 5.2. Apigenin

Apigenin is a flavonoid bioactive compound extracted from citrus fruits. It has presented promising neuroprotective activity in the management of several central nervous system disorders including insomnia and anxiety. Apigenin has also been proven to cross the blood brain barrier and play a neuroprotective role in managing cerebral ischemic reperfusion injuries. This bioflavonoid molecule possesses anti-inflammatory activities and has been demonstrated to play its protective role in the management of various neurodegenerative disorders including Alzheimer’s disease [67]. The kainate model of excitotoxicity in mice also demonstrated significant antioxidant properties of Apigenin and further quenched reactive oxygen species molecules by preventing kainic acid mediated excitotoxicity in the animal groups and the inhibition of glutathione depletion in the hippocampal region of the brain. Several behavioral studies performed in animals indicated the modulation of noradrenergic, dopaminergic, and serotonergic neurons in the brain and demonstrated its anti-depressant effects. A significant modulation in neurotransmitters and an increase in dopamine turnover was also observed in mouse brain upon the administration of apigenin post induction of 40 min of stressful forced swimming, indicating a significant role of dopaminergic neurons as a response to the administration of apigenin. Further exploration into the mechanistic pathways responsible for the anti-depressant effects of apigenin highlighted the inhibition of the monoamine oxidase (MAO) enzyme, preferably MAO A as compared to MAO B, which have been renowned for their anti-depressant activity [68]. Therefore, apigenin has been evaluated to be a potent bioactive molecule useful in the management of depression and conditions of chronic stress.

### 5.3. Amentoflavone

Amentoflavone is a further dimer of apigenin and is a unique bioflavonoid compound. It is mainly isolated from different species of Hypericum and has a tendency to bond with the benzodiazepine binding sites present at gamma aminobutyric acid (GABA) receptors and produces anti-depressant effects [69]. Amentoflavone is extracted by the decoction of *Cnestisferruginea* roots, which have proven anti-depressant activities confirmed after several behavioral tests including a forced swimming test [70].

### 5.4. Chlorogenic Acid

Chlorogenic acid is a renowned antioxidant polyphenolic molecule with neuroprotective capabilities and has been found to comprise neuroprotective properties upon several studies. The biomolecule has been abundantly found in coffee and presents an anxiolytic property which is mainly due to its interaction with GABA-benzodiazepine receptors. The neuroprotective role of chlorogenic acid has been confirmed by several studies performed in animal models of Alzheimer’s disease and cerebral ischemia [71]. The activity of enzyme AchE was also found to be inhibited besides the scavenging of reactive oxygen species upon the administration of chlorogenic acid, which is also responsible for its antidepressant activity. Several preclinical and clinical studies are also underway to explore the activity of this biomolecule in the management of depression and mood elevation [72].

### 5.5. Ellagic Acid

The polyphenolic compound Ellagic acid is mainly found in the stem and bark of eucalyptus, raspberries, pomegranate, and several nuts. The bioflavonoid is a proven antioxidant, anti-inflammatory, and to cancer and cardioprotective molecule and has emerged as a newly antidepressant molecule [73]. Several preclinical studies performed in animal models of depression showed the considerable effects of ellagic acid comparable to the selective serotonin reuptake inhibitor fluoxetine in lieu of its indulgence with monoaminergic neurotransmitter receptors [74,75]. The role of opiodergic receptors has been exempted in the anti-depressant activity of ellagic acid. The role of ellagic acid in major depression has predominantly been confirmed by several preclinical studies, however the clinical studies for the same are underway, and no relevant data has been obtained from the same [76].

### 5.6. Ferulic Acid

**Figure 4:**
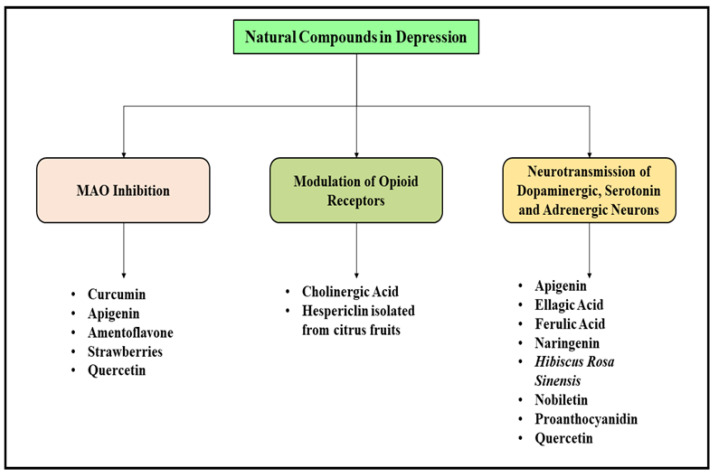
Pathological factors responsible for the initiation and prolongation of depression and list of herbal plants and bioactive natural origin molecules with anti-depressant effects.

Ferulic acid, also known as 4-hydroxy-3-methoxycinnamic acid, is a powerful antioxidant biomolecule and successfully scavenges free radicals from the body. Ferulic acid acts as a potent anti-inflammatory, anticancer, neuroprotective, anti-atherosclerotic and antidiabetic agent and has shown improvement in memory and learning in a study performed in mice induced with dementia. Ferulic acid promotes the neurotransmission of the cholinergic system and antagonizes the excitation of neurotoxic neurons besides reducing oxidative stress. The prolonged consumption of ferulic acid has shown a reduction in the beta-amyloid mediated damage in the brain and hence acts a potent neuroprotective agent [77,78]. Furthermore, the sodium salt of ferulic acid, namely sodium ferulate, has also presented sedative action and has been found to be useful in the management of stasis, headache and irritable mood. Ferulic acid was found to enhance the number of newly generated neuronal cells in the hippocampal region of the brain and prevented neurodegradation and acts as an antidepressant molecule [79]. In several studies performed in depression induced mice, the antidepressant like activity of ferulic acid was found to be significant as it reversed the depressive behavior of the experimental animals upon consumption as observed from the forced swim test. Several other bioactive compounds which possessed significant antidepressant actives have been enlisted in Figure 4.

## 6. Natural Products and Their Protective Role in Management of Ischemic Stroke

Stroke is one of the major leading causes of disability and death globally and hence investigations focusing on the pathogenesis and etiology of the mechanistic pathways responsible for its initiation are undergoing to develop promising strategic treatment approaches which provide neuroprotection with minimum side effects associated with them. The lack of effective therapeutic treatments for the management of stroke has led to an increased interest in the exploration of natural plants and bioactive compounds isolated from them. The bioactive molecules isolated from natural plants provide external stimulants and help in maintenance and restoration of internal balance of the human body. The preventive effects of several natural medicines in stroke have recently been examined and an improvement in the brain microcirculation with simultaneous prevention of ischemic and reperfusion injury in response to reduced oxidative stress was noted, thereby implicating their positive role in the management and prevention of the disease. The production of reactive oxygen species is hastened upon development of ischemic stroke which further induces oxidative stress and damages proteins and DNA due to peroxidation of lipid membrane and eventually causes cell death [80]. The neuroprotective effects of several natural medicines have been confirmed due to their free radical scavenging properties and ultimately the mitigation of oxidative stress and further down streaming of macromolecules thereby implicating further research to explore their therapeutic role in management of neurological and cerebrovascular disorders [81]. The signaling pathways associated with oxidative stress have also been found to be altered upon treatment with bioactive compounds isolated from natural herbal drugs. An acute episode of stroke is followed by extravagation of neuroinflammation which ultimately results into death of neuronal cells. The immune cells release signals for stimulation of proinflammatory cytokines which further activate resident cells and cause infiltration of peripheral inflammatory cells into the lesion area and induces neuroinflammation which is one of the key factors responsible for stroke. The inhibition of the induced inflammatory responses after an acute episode of stroke can significantly prevent injuries in the brain and reduce further long-term outcomes of neurological degenerative conditions. The natural plants and bioactive compounds isolated from them have a tendency to protect the human brain from ischemia-reperfusion injury by mitigating the neuroinflammation which occurs upon the acute stages of stroke [82].

**Figure 5:**
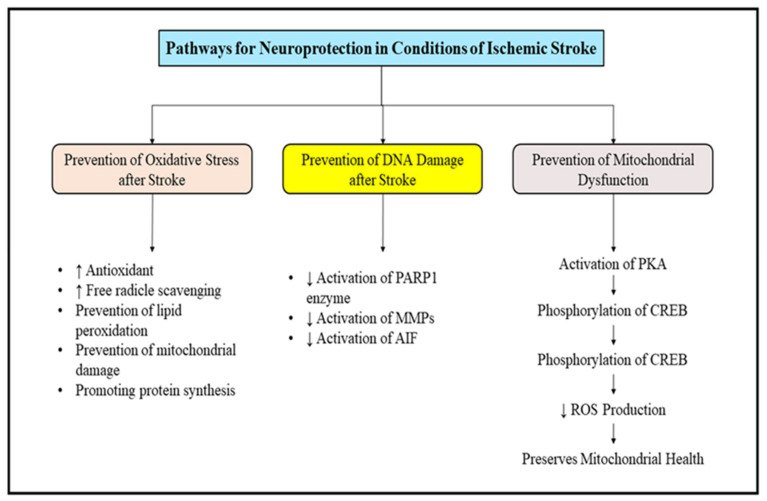
Targeted pathways for inducing neuroprotective effects after ischemic stroke. ROS: Reactive oxygen species; DNA: deoxyribonucleic acids; PARP1: poly ADP-ribose polymerase-1; MMP: Matrix metalloproteinase; AIF: apoptosis inducing factor: PKA: protein kinase A; CREB: cAMP response element-binding protein.

The natural medicines aid in the management of ischemic stroke by decreasing oxidative stress and preventing the damage to DNA and mitochondrial dysfunction by several mechanistic pathways. Oxidative stress due to excessive production of reactive oxygen species (ROS) is the utmost factor responsible for inducing cerebral ischemia and its further progression, which causes injury to brain. It also stimulates the peroxidation of proteins, lipids and nucleic acids which contributes to further dysfunction in mitochondria and produces damage to DNA and ultimately leads to neuronal cell death. After an episode of ischemic stroke, a transient or permanent decrease in the flow of blood is noted; this causes injury to the brain tissue and can even lead to its death [83]. The restoration of blood flow has been proposed to be helpful for the management of ischemic stroke, however, through studies it has also been determined that reperfusion of blood flow induces injury to the brain due to the presence of a large number of ROS which are synthesized during this process, and damage the cellular components including carbohydrates, nucleic acids, proteins and lipids. The ROS interfere with the synthesis of proteins and cause damage to the DNA, mitochondrial structure and endothelial cells, which further reduces the production of energy and causes microcirculation disorders. The impairment of DNA further worsens the neurodegeneration, and its repair is critical to restore normal function and is the primary cause of several neurovascular disorders. Decreasing the activation of poly ADP-ribose polymerase-1 (PARP-1), matrix metalloproteinases and apoptosis inducing factor (AIF) can significantly prevent DNA damage. ROS synthesis and the lipid reoxidation process in the body comprise a dynamic equilibrium disruption which induces a series of metabolic diseases and impairs the functioning of bio membranes of cellular proteins and hence plays a crucial role in the cellular damage produced after an episode of ischemic stroke [84]. The promotion of antioxidant system of body, i.e., activation of superoxide dismutase, catalase and glutathione and further scavenging of ROS is one of the best approaches which can be implemented in the management of oxidative stress mediated brain damage. Mitochondria are the powerhouse of the cell and damage to them is a crucial event that occurs after an episode of ischemic stroke. The excessive amount of ROS generated by mitochondria terminates the electron transport chain and reduces the production of ATP, consequently damaging the functioning of this cellular organelle. The dysfunction of mitochondrial DNA can be prevented by the activation of protein kinase A, which further causes the phosphorylation of CREB and decreases the production of ROS [85]. To conclude, mitochondrial dysfunction has been evaluated to be a prominent factor involved in the pathology of several neurodegenerative disorders, thereby indicating it to be a promising target for the development of therapeutic treatments for management of neurodegenerative disorders. The compiled descriptions of pathways which can be targeted for the management of ischemic stroke are described in Figure 5.

**Figure 6:**
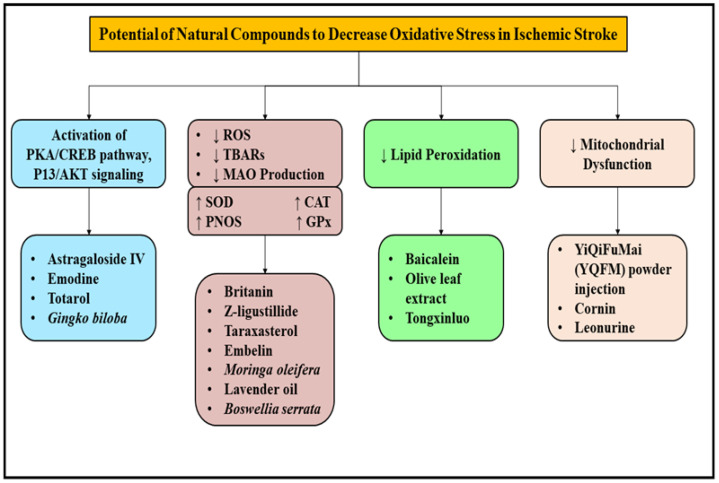
Herbal plants and natural origin bioactive compounds and their mechanisms involved in the management of Ischemic stroke. ROS: Reactive oxygen species; PKA: protein kinase A; CREB: cAMP response element-binding protein; PI3/AKT: phosphatidylinositol 3-kinase/protein kinase B; TBARS: Thiobarbituric acid reactive substances; MAO: Mono amino oxidase; SOD: sodium dismutase; CAT: catalase; GPx: glutathione.

Totarol, isolated from *Podocarpus totora*, has been studied to reduce the volume of infarct in in-vivo and in-vitro studies and was concluded to attenuate the effects of ischemia-reperfusion injury. It mainly acts by increasing the antioxidant levels of glutathione and superoxide dismutase and elevating the levels of heme oxygenase 1 and other associated proteins by activating the PKA/Creb pathway, thereby suppressing the levels of ROS and producing neuroprotective effects [86]. Taraxasterol, a pentacyclic triterpene namely extracted from *Taraxacum officinale*, also reduced the production of ROS in the hippocampal region of the brain, which further ameliorated oxidative stress by regulating the Nrf2 signaling pathway and prevented ischemic injury [87]. Britanin, an activator of Nrf2 protein, also displayed neuroprotective effects and presented excellent protection towards the cerebrum in an animal model induced with middle cerebral artery occlusion and reperfusion. The antioxidant activity of britanin was mainly due to its selective binding with the 151 conserved cysteine residue of Keap1 and prohibited the ubiquitylation of Nrf2 and hence prevented ischemic injury [88]. A major bioactive compound isolated from the dried roots of Astragalus, species namely Astragaloside IV, was found to improve cognition and memory impairment resulting from ischemic stroke. The molecule successfully protected the neurons from further injury and death upon an acute episode of ischemic stroke by maintaining the functioning of mitochondria via modulating the PKA/CREB pathway [89]. Several natural medicines with antioxidant activity and the potential to improve mitochondrial dysfunction are being extensively researched and used in the management of countless neurodegenerative disorders. Leonurine and cornin, other antioxidant natural bioactive compounds, have been indicated to decrease the volume of infarct and improve neurological defects by modulating the functioning of mitochondria and prohibiting the production of ROS and further suppressing oxidative stress [90,91]. A bioactive molecule isolated from *Tripterygium wilfordii*, namely celastrol, protected the cerebral region of the brain from an acute episode of cerebral ischemia by suppressing oxidative stress via phosphorylation of the c-Jun-N-terminal kinase (p-JNK) and NF-κB which aided in the reduction of ROS in the brain [92]. Emodin, a bioactive compound extracted from *Polygonum multiforum* considerably decreased the volume of infarct by activating the signaling of phosphatidylinositol 3-kinase (PI3K)/protein kinase B (AKT), which considerably improved the functioning of motor functions of the brain and prevented damage induced by ischemic stroke [93]. Another bioactive benzoquinoe derivative, namely Z-ligustilide, which is isolated from *Angelica sinensis*, protected the disruption of blood brain barrier by alleviating neuroinflammation and oxidative stress after an acute episode of ischemic stroke [94]. Embelin, a naturally occurring bioactive molecule derived from dried berries of *Embelicaribes*, produced significant recovery of motor functions of the brain and presented protective effects by reducing the levels of lipid peroxidase and increasing glutathione content, thereby expressing antioxidant activity and significantly preventing the after effects of ischemic stroke [95]. Hence, a wide variety of natural medicines have been studied and explored in the management of ischemic stroke, as shown in Figure 6, which implicates natural medicines to be an emerging and promising treatment approach for the management of damage induced by ischemic stroke and reperfusion injury.

## 7. Challenges, Future Prospects and Conclusions

The ability of natural products to express neuroprotective effects have been well established by several researchers and the literature verifying it is significant. Notably, several biomolecules originating from herbal and natural sources which are being used in management of wide array of diseases and which have reported numerous adverse effects have also been discovered. An example is ephedra, a commonly used herbal molecule isolated from *Ephedra sinica*, which is extensively used in severe respiratory conditions such as cough and wheezing, although it’s excessive use has resulted in serious toxicities including neurological conditions such as psychosis and seizures, and cardiovascular disorders including arrythmias, myocardial infarction, stroke and anticholinergic poisoning [96]. Several other molecules including strychnine, thujone and essential oils such as camphor and eucalyptus have also been reported to induce seizures upon their excessive consumption. The species of Valeriana and kava kava also possess a tendency to induce sedation [97]. Several other commonly used herbal plants such as Opium (*Papaver somniferum*) used in the management of chronic pain, Vinca (*Catharanthus roseus*), used in treatment of cancers, diabetes mellitus, marijuana (*Cannabis indica*), used in management of nerve pain and commonly as a recreational drug, Thorn apple (*Datura stramonium*), as an anti-inflammatory, anti-infective agent, Deadly nightshade (*Atropa belladonna*),used in management of respiratory and intestinal conditions, etc., possess neurotoxic effects when administered in large doses [98]. With the progression of time and technology, the development of products and tools which aid in the extraction of herbal drugs and further isolation of bio active molecules from them is also advancing; this has led to the exploration of several natural drug moieties which produce successful therapeutic target effects with minimal adverse events. Despite positive results collected through preclinical studies, the natural products and biomolecules isolated from them face several challenges such as poor bioavailability, low solubility and absorption, rapid metabolism, instable chemical structures and poor affinity towards the blood brain barrier [99]. For instance, the most effective natural bioactive molecules, namely curcumin and resveratrol, have been reported to possess a low level ability and degrade or transform into inactive compounds easily due to their unstable molecules thereby reducing their efficacy. The blood brain barrier also acts as a shield and prevents the penetration of natural bio active molecules into the site of action in the brain, thereby limiting their distribution into brain tissues and lowering their therapeutic effects. Thus, efforts are being made to overcome these challenges by nanotechnology and nanocarrier formulations which will significantly prove to be effective in delivering natural drug molecules to the target site of action in the brain and produce significant therapeutic effects with minimal adverse events. The formulation of natural isolated components into nanoparticles as a drug delivery approach can prove to be fruitful in improving the bioavailability of these compounds and hence increasing their absorption in the body and preventing their drastic biotransformation into inactive molecules, thereby amplifying their therapeutic effects. The natural products and biotic molecules isolated from them are needed to manage and prevent several neurodegenerative disorders and producing least notable adverse events. Based on the multi-pathway action of natural medicines, they have a potential to target molecules and pathologies involved in the initiation and progression of neurodegeneration, thereby presenting neuroprotection and a promising treatment approach. For effective management of the neurological disorders, it is essential that the natural products and their biomolecules successfully penetrate the blood brain barrier and reach the target site of action [100]. To accomplish and overcome the challenges of natural products, advanced nanotechnological techniques and drug delivery approaches are being explored to enhance the therapeutic potential of the natural products and deliver neuroprotective effects with minimal toxicity.

## Data Availability

Not applicable.

## Abbreviations

Ach	Acetylcholine
AchE	Acetylcholinesterase
AICD	APP intracellular domain
AIF	apoptosis inducing factor
APP	amyloid precursor protein
ATP	Adenosine triphosphate
Aβ	Amyloid beta
BACE-1	β-secretase
BDNF	Brain derived neurotrophic factors
BuChE	Butyrylcholinesterase
CAG	cytosine-adenine-guanine
cAMP	cyclic adenosine monophosphate
ChAT	O-acetyltransferase
COX	cyclooxygenase
CREB	cAMP response element-binding protein
CRP	C reaction protein
DNA	deoxy ribonucleic acids
EGCG	Epigallocatechin-3-gallate
GABA	gamma aminobutyric acid
HDAC	Histone deacetylase
IL-1β	interleukin 1 β
MAO A	Monoamine oxidase A
MAO B	Monoamine oxidase B
MAO	Monoamine oxidase
MDA	malondialdehyde
MMP	Matrix metalloproteinase
mRNA	messenger ribonucleic acid
nAchR	nicotinic acetylcholine receptors
NF-ĸB	Nuclear factor kappa B
NO	Nitric oxide
PARP 1	poly ADP-ribose polymerase-1
PI3K	phosphatidylinositol 3-kinase
p-JNK	c-Jun-N-terminal kinase
PKA	protein kinase A
ROS	reactive oxygen species
sAPPβ	soluble amyloid precursor proteins
TNF-α	Tumor necrosis factor-α
